# -866G/A and Ins/Del polymorphisms in the *UCP2* gene and diabetic kidney disease: case-control study and meta-analysis

**DOI:** 10.1590/1678-4685-GMB-2018-0374

**Published:** 2020-03-27

**Authors:** Cristine Dieter, Taís Silveira Assmann, Natália Emerim Lemos, Eloísa Toscan Massignam, Bianca Marmontel de Souza, Andrea Carla Bauer, Daisy Crispim

**Affiliations:** 1 Hospital de Clínicas de Porto Alegre, Endocrine Division, Porto Alegre, RS, Brazil.; 2 Universidade Federal do Rio Grande do Sul, Faculdade de Medicina, Programa de Pós-Graduação em Ciências Médicas: Endocrinologia, Porto Alegre, RS, Brazil.; 3 Universidad de Navarra, Department of Nutrition, Food Science and Physiology, Pamplona, Spain.; 4 Hospital de Clínicas de Porto Alegre, Nephrology Division, Porto Alegre, RS, Brazil.

**Keywords:** UCP2, polymorphisms, diabetic kidney disease

## Abstract

Uncoupling protein 2 (UCP2) decreases reactive oxygen species (ROS). ROS overproduction is a key contributor to the pathogenesis of diabetic kidney disease (DKD). Thus, *UCP2* polymorphisms are candidate risk factors for DKD; however, their associations with this complication are still inconclusive. Here, we describe a case-control study and a meta-analysis conducted to investigate the association between *UCP2* -866G/A and Ins/Del polymorphisms and DKD. The case-control study comprised 385 patients with type 1 diabetes mellitus (T1DM): 223 patients without DKD and 162 with DKD. *UCP2* -866G/A (rs659366) and Ins/Del polymorphisms were genotyped by real-time PCR and conventional PCR, respectively. For the meta-analysis, a literature search was conducted to identify all studies that investigated associations between *UCP2* polymorphisms and DKD in patients with T1DM or type 2 diabetes mellitus. Pooled odds ratios were calculated for different inheritance models. Allele and genotype frequencies of -866G/A and Ins/Del polymorphisms did not differ between T1DM case and control groups. Haplotype frequencies were also similar between groups. Four studies plus the present one were eligible for inclusion in the meta-analysis. In agreement with case-control data, the meta-analysis results showed that the -866G/A and Ins/Del polymorphisms were not associated with DKD. In conclusion, our case-control and meta-analysis studies did not indicate an association between the analyzed *UCP2* polymorphisms and DKD.

## Introduction

**Figure 1:**
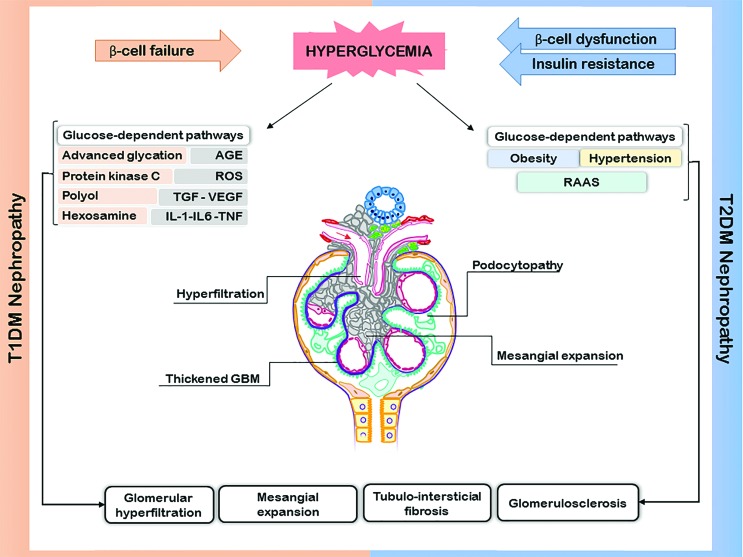
DKD pathogenesis in T1DM and T2DM. Chronic hyperglycemia has a central role in the pathophysiology of DKD. In T1DM, chronic hyperglycemia activates several known pathways associated with the development and progression of the diabetic nephropathy, to cite: advanced glycation, polyol, hexosamine and protein kinase C pathways. In T2DM, besides these pathways, the presence of obesity and/or hypertension through hemodynamic mechanisms activates the renin-angiotensin-aldosterose system (RAAS), leading to glomerular hyperfiltration. All these factors participate in the pathophysiology of the DKD, characterized by the thickness of the glomerular basement membrane (GBM), podocytopathy, mesangial expansion and glomerulosclerosis, and that are the key mechanisms to diabetic nephropathy. AGE: advanced glycation end-products; ROS: reactive oxygen species; TGF: transforming growth factor; VEGF: vascular endothelial growth factor; IL: interleukin; TNF: tumor necrosis factor.

Diabetic kidney disease (DKD) is a common microvascular complication that affects 40% of patients with diabetes mellitus (DM) (Gross *et al.*, 2005, Macisaac *et al.*, 2014). DKD is the leading cause of end-stage renal disease in subjects starting renal replacement therapy and is associated with increased cardiovascular mortality (Gross *et al.*, 2005, Assmann *et al.*, 2018). This complication is a progressive disease, characterized by pathophysiological changes resulting from the diabetic milieu, which begin with glomerular hypertrophy and hyperfiltration, and might progress to albuminuria and a gradual decline in the glomerular filtration rate (GFR) (Kanwar *et al.*, 2011, Ritz *et al.*, 2011). The progressive decline in renal function during DKD is a result of pathophysiological alterations in the kidneys, such as mesangial expansion and tubulointerstitial fibrosis due to the accumulation of extracellular matrix proteins, basement membrane thickening, and podocyte dysfunction (Assmann *et al.*, 2018) (Figure 1). The main risk factors for DKD are the duration of chronic hyperglycemia, arterial hypertension, dyslipidemia, and genetic susceptibility (Carpena *et al.*, 2010, Ahlqvist *et al.*, 2015).

At the cellular level, chronic hyperglycemia causes renal damage through five main mechanisms: increased formation of advanced glycation end-products; increased expression of the receptor for advanced glycation end-products; activation of protein kinase C isoforms; increased flux of glucose through the polyol pathway; and upregulation of the hexosamine pathway (Du *et al.*, 2000, Giacco and Brownlee, 2010). Several lines of evidence have shown that the mitochondrial overproduction of reactive oxygen species (ROS) is the unifying upstream mechanism by which hyperglycemia activates all these five pathways (Brownlee, 2005; Rich, 2006; Giacco and Brownlee, 2010).

Uncoupling protein 2 (UCP2) is a mitochondrial anion carrier protein expressed in a number of tissues, including adipose tissue, liver, kidney, and retina (Souza *et al.*, 2011; Donadelli *et al.*, 2014). This protein mildly uncouples the oxidative phosphorylation from ATP synthesis by dissipating the proton gradient generated across the mitochondrial inner membrane; thereby, decreasing ATP production. The uncoupling then leads to tissue-specific functions, such as regulation of glucose and lipid metabolism and immune cell activation and, importantly, reducing ROS formation by mitochondria (Souza *et al.*, 2011; Toda and Diano, 2014).

Consistent with the role of UCP2 in decreasing oxidative stress, several studies have suggested that polymorphisms in the *UCP2* gene are associated with ROS-related pathologies (Ji *et al.*, 2004; Yu *et al.*, 2009; Chai *et al.*, 2012) and with the development of DM and its chronic complications (Jia *et al.*, 2009; Crispim *et al.*, 2010; de Souza *et al.*, 2012, 2013, 2015). To date, three common *UCP2* polymorphisms have been well studied: the functional -866G/A polymorphism (rs659366) in the promoter region; the Ala55Val polymorphism (rs660339) in exon 4, and the 45 bp insertion/deletion (Ins/Del) polymorphism in the 3’ untranslated region (Jia *et al.*, 2009; Dalgaard, 2011).

Our group previously showed that the polymorphic *UCP2* -866A/55Val/Ins haplotype (constituted by the -866G/A, Ala55Val, and Ins/Del polymorphisms) was associated with risk for proliferative diabetic retinopathy (DR) in type 1 and type 2 diabetic patients (Crispim *et al.*, 2010). The -866G/A and the Ala55Val polymorphisms were in almost complete linkage disequilibrium in our population from the South of Brazil (Crispim *et al.*, 2010). Recently, we reported that the polymorphic -866A/55Val/Ins haplotype was also an independent risk factor for DKD (OR = 2.14, 95% CI 1.04 – 4.40) in patients with type 2 diabetes mellitus (T2DM) (de Souza *et al.*, 2015). Moreover, T2DM patients carrying the polymorphic haplotype showed lower estimated GFR compared with patients carrying the reference haplotype (-866G/Ala55/Del). Interestingly, the polymorphic haplotype was associated with decreased *UCP2* gene expression in human kidney biopsy samples (de Souza *et al.*, 2015).

Therefore, here we performed a case-control study to investigate if the *UCP2* -866G/A and Ins/Del polymorphisms were also associated with DKD in patients with type 1 diabetes mellitus (T1DM). Additionally, we conducted a systematic review and meta-analysis of the literature on the subject as part of the ongoing effort to evaluate if *UCP2* polymorphisms are associated with DKD in T1DM or T2DM patients.

## Subjects and Methods

### Case-control study

#### Subjects, phenotype measurements, and laboratory analyses

This case-control study was designed in agreement with STROBE and STREGA guidelines for reporting of genetic association studies (von Elm *et al.*, 2008; Little *et al.*, 2009). The sample population comprised 162 T1DM patients with DKD (cases) and 223 T1DM patients without this complication and with at least 10 years of DM duration (T1DM controls). All T1DM patients were recruited from the outpatient clinic at Hospital de Clínicas de Porto Alegre (Rio Grande do Sul, Brazil). Patients were diagnosed as having T1DM according to American Diabetes Association guidelines (American Diabetes Association, 2018). A standard questionnaire was used to collect information on age, age at T1DM diagnosis, T1DM duration, and drug treatment. In addition, all patients underwent physical and laboratory evaluations, as previously described (Boucas *et al.*, 2013; Assmann *et al.*, 2014). The ethnic group was defined based on self-classification.

Serum and plasma samples were taken after 12 h of fasting for laboratory analyses (Boucas *et al.*, 2013; Assmann *et al.*, 2014). Glucose levels were determined using the glucose oxidase method. Glycated hemoglobin (HbA1c) levels were measured by different methods and the results were traceable to the Diabetes Control and Complications Trial method by off-line calibration or using a conversion formulae (Camargo *et al.*, 1998). Creatinine was measured by the Jaffé reaction; total plasma cholesterol, HDL cholesterol and triglycerides by enzymatic methods, and albuminuria by immunoturbidimetry (Sera-Pak immuno microalbuminuria, Bayer, Tarrytown, NY, USA) (Zelmanovitz *et al.*, 1997).

The diagnosis of DKD was based on the urinary albumin excretion (UAE) in at least two out of three consecutive 24 h timed urine samples in a 6-month period. Patients were classified as having normal to mildly increased UAE (UAE < 30 mg / 24 h, control group), moderately increased UAE (UAE 30 – 299 mg / 24 h) or severely increased UAE (UAE > 300 mg / 24 h) (KDIGO Group, 2013). Therefore, the case group comprised patients who had moderately to severely increased UAE (moderate to severe DKD). Patients with other causes of albuminuria or renal diseases were excluded from the study. The estimated GFR was calculated using the Chronic Kidney Disease Epidemiology Collaboration (CKD-EPI) equation: estimated GFR = 141 x min (SCR/κ, 1)^a^ x max (SCR/κ, 1)^-1,209^ x 0,993^age^ x 1,018 [if female] x 1,159 [if black] (Levey *et al.*, 2009).

In addition, we also included a third group constituted of 489 healthy blood donors recruited from the same hospital, and who did not have diabetes or family history of this disease. These subjects were used as non-diabetic controls; thus, only subjects with HbA1c < 5.7% were included in this group (American Diabetes Association, 2018). All subjects gave assent and written informed consent prior to participation. The study protocol was approved by Ethic Committee in Research from Hospital de Clínicas de Porto Alegre.

#### Genotyping

DNA was extracted from peripheral blood leucocytes by a standardized salting-out procedure (Lahiri and Nurnberger, 1991). *UCP2* -866G/A polymorphism (rs659366) was genotyped using primers and probes contained in the TaqMan SNP Genotyping Assay 20 (Thermo Fisher Scientific, Foster City, CA, USA – assay ID: C___8760350_10). Real-Time PCR reactions were performed in 384 well plates, in a total 5 μL volume, using 2 ng of DNA, TaqMan Genotyping Master Mix 1 (Thermo Fisher Scientific) and TaqMan Genotyping Assay 1. The assays were done in a real-time PCR thermal cycler (ViiA7 Real-Time PCR System; Thermo Fisher Scientific) with the following protocol: heating for 10 min at 95 °C, followed by 50 cycles of 95 °C for 15 s and 62 °C for 90 s. Genotyping of the *UCP2* 45 bp Ins/Del polymorphism was performed by direct separation of the PCR products on 2.5% agarose gel stained with GelRed , as previously described (de Souza *et al.*, 2012).

As the -866G/A polymorphism is in almost complete linkage disequilibrium with the Ala55Val polymorphism (|D’| = 0.991, r^2^ = 0.905) in our population, only the *UCP2* -866G/A and Ins/Del polymorphisms were analyzed in the present case-control study (Crispim *et al.*, 2010).

#### Statistical analyses for the case-control study

Allele frequencies were determined by gene counting, and departures from the Hardy-Weinberg Equilibrium were verified using the χ^2^ test. Allele and genotype frequencies were compared between groups of subjects using χ^2^ tests. Between all pairs of biallelic loci, we examined widely used measures of linkage disequilibrium, Lewontin’s D’ |D’| and r^2^ (Hedrick, 1987). Haplotypes constructed with the combination of the two *UCP2* polymorphisms and their frequencies were inferred using the PHASE 2.1 program, which implements a Bayesian statistical method (Stephens *et al.*, 2001).

Clinical and laboratory characteristics were compared between group of patients categorized according to the different genotypes of the two *UCP2* polymorphisms using unpaired Student’s *t* test, One-Way ANOVA or χ^2^ test, as appropriate. Variables with normal distribution are shown as mean ± SD or percentage. Variables with skewed distribution were log-transformed before analysis and are shown as median (25^th^ – 75^th^ percentile values). Multivariate logistic regression analyses were done to evaluate the independent association of each individual *UCP2* polymorphism or haplotypes with DKD, adjusting for possible confounding factors. Variables with significant associations with DKD in the univariate analysis, or with an important biological association with this complication were chosen for inclusion in the multivariate model. T1DM duration was not included as an independent variable in these analyses since T1DM control group was selected based on this characteristic. Statistical analyses were performed using the SPSS 18.0 software (SPSS, Chicago, IL), and *P*-values < 0.05 were considered significant.

### Systematic review and meta-analysis

#### Search strategy and eligibility criteria

This study was designed and reported in accordance with the Preferred Reporting Items for Systematic Reviews and Meta-Analyses (PRISMA) and Meta-analysis of Observational Studies in Epidemiology (MOOSE) statements (Stroup *et al.*, 2000; Moher *et al.*, 2009). PubMed and Embase repositories were searched to retrieve all articles that investigated associations between DKD and at least one of the two polymorphisms of interest. The Medical Subject Headings used for this search are shown in Supplementary Material – MeSH terms. The search was restricted to human studies and English, Portuguese, or Spanish language articles, and was completed on December, 2018. References from all articles identified were searched manually to find other relevant studies.

Eligibility evaluation was made by title and abstracts review, and when abstracts did not provide adequate information, the full text of the paper was retrieved for evaluation. This was done independently in a standardized manner by two investigators (C.D. and N.E.L.), as previously described (de Souza *et al.*, 2013; Brondani *et al.*, 2014). Discrepancies were solved by discussion between them and, when necessary, a third reviewer (D.C.) was accessed. Observational studies that compared the -866G/A or Ins/Del polymorphisms between patients with and without DKD were included in the meta-analysis. Articles were excluded from the analysis if genotype frequencies in the control group deviated from those predicted by the Hardy-Weinberg Equilibrium, or if they did not have enough data to estimate an OR with 95% CI. If results were duplicated and had been published more than once, the most complete study was chosen.

#### Data extraction and quality control assessment

Necessary information from each study was independently extracted by two investigators (C.D. and N.E.L.) using a standardized extraction form (de Souza *et al.*, 2013; Brondani *et al.*, 2014), and consensus was sought in all extracted items. When consensus could not be achieved, differences in data extraction were decided by reading the original publication or by consulting a third reviewer (D.C.). Data extracted from each study was as follows: (1) characteristics of each study and its samples (including name of the first author, publication year, number of subjects in case and control groups, mean age, gender, ethnicity, and age at T1DM or T2DM diagnosis); (2) case and control definitions; (3) polymorphism frequencies and OR (95% CI). When data were not available, the authors were contacted by e-mail.

Two investigators (C.D. and N.E.L.) independently evaluated the quality of each selected study using the Newcastle-Ottawa Scale (NOS) (Stang, 2010). The NOS contains eight items divided into three dimensions: selection, comparability, and exposure. For each item, a sequence of answer options is provided. A star scoring system is used to allow a semi-quantitative evaluation of paper quality, such that the highest quality studies are given a maximum of one star for each item, with exception of the item related to comparability, which allows two stars to be given. Therefore, the final NOS score varies from 0 to 9 stars.

#### Statistical analysis for meta-analysis

Genotype distributions in control groups were tested for conformity with the Hardy-Weinberg Equilibrium using a goodness-of-fitness χ^2^ test. Associations between polymorphisms and DKD were analyzed using OR (95% CI) calculations based on allele contrast, dominant, recessive and additive inheritance models (Minelli *et al.*, 2005). Heterogeneity was tested using χ^2^-based Cochran’s Q statistic and inconsistency was assessed with the I^2^ metric (Higgins and Thompson, 2002; Higgins *et al.*, 2003). Heterogeneity was considered statistically significant at *P* &λτ; 0.10 for the Q statistic and/or I^2^ &γτ; 50% for the I^2^ statistic. Where significant heterogeneity was detected, the DerSimonian and Laird random effect model (REM) was used to calculate OR (95% CI) for each study and for the pooled effect; where heterogeneity was not significant, the fixed effect model was used. Sensitivity analyses were performed to recognize important studies with a considerable impact on inter-study heterogeneity. All statistical analyses were performed using Stata 11.0 software (StataCorp, College Station, TX, USA).

## Results

### Case-control study

Comparisons of clinical and laboratorial characteristics between T1DM case and control groups, categorized according to UAE values, are show in Table 1. As expected, HbA1c, triglycerides, total cholesterol, LDL cholesterol, and creatinine levels were increased in patients with DKD compared to T1DM controls. Prevalence of arterial hypertension and DR were also increased in the DKD group. Estimated GFR was decreased in patients with DKD compared to T1DM controls. The ethnic proportion did not differ significantly between case and control groups: 10.5% of black subjects in the case group *vs*. 5.4% of black subjects in the control group (*P* = 0.093). Frequencies of the minor alleles of the -866G/A and Ins/Del polymorphisms in white and black subjects were: 40.5% *vs.* 44.8% for the -866A allele (*P* = 0.814), and 30.7% *vs.* 20.3% for the Ins allele (*P* = 0.386).

**Table 1:** Clinical and laboratory characteristics of T1DM patients with UAE > 30 mg/24 h (DKD cases) and T1DM patients with UAE < 30 mg/24 h (T1DM controls).

Characteristics	T1DM controls	DKD cases	P*
	(n = 223)	(n = 162)	
Age (years)	36.8 ± 12.8	37.7 ± 13.6	0.478
Gender (% male)	47.5	48.8	0.892
Ethnicity (% black)	5.4	10.5	0.093
HbA1c (%)	8.4 ± 1.7	9.5 ± 2.2	0.0001
BMI (kg/m^2^)	24.2 ± 3.6	23.9 ± 3.6	0.413
Hypertension (%)	31.8	46.0	0.012
Age at diagnosis (years)	15.4 ± 10.0	15.4 ± 10.6	0.993
T1DM duration (years)	20.7 ± 8.2	20.6 ± 10.5	0.956
Systolic BP (mmHg)	121.1 ± 15.7	123.4 ± 19.3	0.244
Diastolic BP (mmHg)	77.2 ± 10.6	78.3 ± 13.5	0.423
Triglycerides (mg/dL)	70.0 (51.7 – 98.5)	100.0 (70.2 – 159.5)	< 0.001
Total cholesterol (mg/dL)	177.7 ± 42.1	193.0 ± 58.0	0.007
LDL cholesterol (mg/dL)	100.8 ± 30.6	111.5 ± 48.0	0.031
HDL cholesterol (mg/dL)	57.7 ± 16.7	56.2 ± 19.0	0.429
Diabetic retinopathy (%)	44.8	66.9	< 0.001
Serum creatinine (μg/dL)	0.9 (0.7 – 1.0)	1.0 (0.8 – 1.6)	< 0.001
eGFR (ml/min/1.73m^2^)	104.0 (87.2 – 121.0)	87.0 (46.0 – 117.0)	< 0.001
UAE (mg/g)	5.5 (3.3 – 10.7)	86.9 (39.0 – 353.8)	-

Data are shown by mean ± standard deviation, median (25^th^ – 75^th^ percentile values) or %. BMI: body mass index; BP: blood pressure; DKD: diabetic kidney disease; eGFR: estimated glomerular filtration rate; HbA1c: glycated hemoglobin; T1DM: type 1 diabetes mellitus; UAE: urinary albumin excretion. *P-values were calculated using Student’s *t*-tests, or Chi-square tests, as appropriate.

Table 2 shows genotype and allele frequencies of the -866G/A and Ins/Del polymorphisms in T1DM patients with UAE > 30 mg / 24h (DKD cases) and T1DM patients with UAE < 30 mg / 24h (T1DM controls). Genotype distributions of the two analyzed polymorphisms were in agreement with those predicted by the Hardy-Weinberg Equilibrium in both groups (*P* ≥ 0.05), and they were similar between DKD cases and T1DM controls (Table 2). Of note, this result did not change after adjustment for ethnicity, HbA1c, serum creatinine, and triglycerides (Table 2). Accordingly, allele distributions of the -866G/A and Ins/Del polymorphisms did not differ between case and control groups, and these polymorphisms were also not associated with DKD when assuming different genetic inheritance models (Table 2). It is worth of note that when we stratified patients according to the UAE severity (T1DM controls *vs*. patients with moderate UAE *vs*. severe UAE), -866G/A and Ins/Del frequencies also did not differ significantly among groups (Table S1).

**Table 2:** Genotype and allele frequencies of *UCP2* -866G/A and Ins/Del polymorphisms in T1DM patients with UAE > 30 mg/24h (DKD cases) and in T1DM patients with UAE < 30 mg/24h (T1DM controls).

Polymorphisms	T1DM controls	DKD cases	OR (95% CI)/Unadjusted *P-* value*	Adjusted OR (95% CI)/† *P-* value
**-866G/A**	n = 223	n = 162		
Genotype				
G/G	77 (34.5)	61 (37.7)	1	1
G/A	107 (48.0)	72 (44.4)	0.849 (0.542 - 1.332)/ 0.477	0.779 (0.447 – 1.359)/ 0.379
A/A	39 (17.5)	29 (17.9)	0.939 (0.522 – 1.687)/ 0.832	1.263 (0.628 – 2.541)/ 0.513
Allele				
G	0.59	0.60	0.706	-
A	0.41	0.40		
Recessive model				
G/G + G/A	184 (82.5)	133 (82.1)	1	1
A/A	39 (17.5)	29 (17.9)	1.029 (0.606 – 1.747)/ 0.917	1.449 (0.771 – 2.723)/ 0.249
Additive model				
G/G	77 (66.4)	61 (67.8)	1	1
A/A	39 (33.6)	29 (32.2)	0.939 (0.522 – 1.687)/ 0.832	1.313 (0.634 – 2.717)/ 0.463
Dominant model				
G/G	77 (34.5)	61 (37.7)	1	1
G/A + A/A	146 (65.5)	101 (62.3)	0.873 (0.573 – 1.330)/ 0.528	0.900 (0.538 – 1.506)/ 0.689
**Ins/Del**	n = 222	n = 156		
Genotype				
Del/Del	107 (48.2)	82 (52.6)	1	1
Ins/Del	93 (41.9)	59 (37.8)	0.828 (0.536 – 1.279)/ 0.394	0.710 (0.411 – 1.225)/ 0.219
Ins/Ins	22 (9.9)	15 (9.6)	0.890 (0.435 – 1.822)/ 0.749	1.453 (0.616 – 3.551)/ 0.393
Allele				
Del	0.69	0.71	0.492	-
Ins	0.31	0.29		
Recessive model				
Ins/Del + Del/Del	200 (90.1)	141 (90.4)	1	1
Ins/Ins	22 (9.9)	15 (9.6)	0.967 (0.485 – 1.930)/ 0.924	1.705 (0.749 – 3.881)/ 0.204
Additive model				
Del/Del	107 (82.9)	82 (84.5)	1	1
Ins/Ins	22 (17.1)	15 (15.5)	0.890 (0.435 – 1.822)/ 0.749	1.276 (0.545 – 2.990)/ 0.574
Dominant model				
Del/Del	107 (48.2)	82 (52.6)	1	1
Ins/Del + Ins/Ins	115 (51.8)	74 (47.4)	0.840 (0.557 – 1.265)/ 0.403	0.821 (0.493 – 1.367)/ 0.448
**Presence of the** *UCP2* mutated haplotype	(n = 209)	(n = 150)		
0 or 1 mutated allele	110 (52.6)	83 (55.3)	1	1
2 mutated alleles	63 (30.2)	44 (29.4)	0.926 (0.573 – 1.494)/ 0.752	0.751 (0.411 – 1.372)/ 0.352
3 or 4 mutated alleles	36 (17.2)	23 (15.3)	0.847 (0.467 – 1.536)/ 0.584	1.207 (0.593 – 2.458)/ 0.604

Data are shown as number (%) or proportion. DKD: diabetic kidney disease; T1DM: type 1 diabetes mellitus; UAE: urinary albumin excretion. *P-values were calculated using Chi-square tests. † *P*-values and OR (95% CI) obtained using logistic regression analyses adjusting for ethnicity, HbA1c, serum creatinine (logarithmic scale), and triglycerides (logarithmic scale).

The -866G/A polymorphism is in moderate linkage disequilibrium with the Ins/Del polymorphism (|D’|= 0.711, r^2^= 0.311) in our population. Four haplotypes (Ht) produced by the combination of these two polymorphisms were inferred in the total sample of T1DM patients: -866G/Del (Ht1; 52.7%), -866A/Del (Ht2; 17.2%), -866G/Ins (Ht3; 6.5%) and -866A/Ins (Ht4; 23.6%). Distributions of these haplotypes were similar between T1DM controls and cases with DKD (*P* = 0.892) (Table 3). Moreover, frequency of 3 or 4 minor alleles of the -866G/A and Ins/Del polymorphisms (Ht3/Ht4 or Ht4/Ht4) were similar between T1DM controls and patients with DKD (17.2% *vs*. 15.3%, adjusted *P* = 0.604; Table 2). These frequencies were also similar among groups according to the severity of DKD (T1DM controls *vs*. moderate UAE *vs*. severe UAE; *P* = 0.805; Table S1).

**Table 3:** Haplotypes of the *UCP2* polymorphisms in T1DM patients with and without DKD.

Haplotypes	T1DM controls (n = 418)	DKD cases (n = 300)	*P*-value
Ht 1 (-866G/Del)	0.518	0.540	
Ht 2 (-866A/Del)	0.171	0.173	0.892
Ht 3 (-866G/Ins)	0.069	0.060	
Ht 4 (-866A/Ins)	0.242	0.227	

Data are presented as proportion. n = number of chromosomes. DKD: diabetic kidney disease; T1DM: type 1 diabetes mellitus. The first letter of the haplotypes refers to the -866G/A polymorphism and the second to the Ins/Del polymorphism. *P*-values for the comparisons of haplotype frequencies between patients with or with DKD were calculated using permutations tests.

In an exploratory analysis, all clinical and laboratory characteristics showed in Table 1 were then compared between all T1DM patients (control + case subjects) broken down by the presence of the -866G/A and Ins/Del polymorphisms. The frequency of DR was not significantly different among -866G/A genotypes (G/G: 49.2%; G/A: 53.8% and A/A: 65.1%; *P* = 0.117). In contrast, presence of DR was increased in patients carrying the Ins/Ins genotype (81.8%) compared to patients with the Del/Del or Ins/Del genotypes (48.5% and 57.0%, respectively; *P* = 0.002). Frequency of DR was 70.4% in patients carrying 3 or 4 minor alleles of the -866G/A and Ins/Del polymorphisms, 49.7% in patients with 0/1 minor allele, and 54.1% in patients with 2 minor alleles (*P* = 0.029). No other characteristic described in Table 1 differed among the genotypes of the two analyzed polymorphism (data not shown). Genotype and allele frequencies of the -866G/A and Ins/Del polymorphisms were similar between T1DM patients (T1DM controls + DKD patients) and non-diabetic subjects (Table S2), suggesting that these two polymorphisms are not associated with T1DM risk.

### Systematic review and meta-analysis

**Figure 2:**
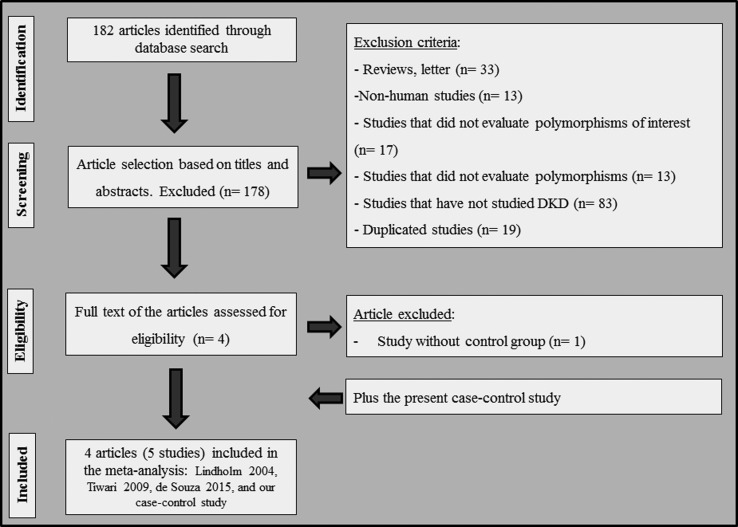
Flow diagram illustrating the search strategy used to identify association studies of *UCP2* polymorphisms and DKD for inclusion in the meta-analysis study.

Figure 2 shows a flow diagram illustrating the strategy used to identify and select articles for inclusion in our meta-analysis. A total of 182 possible relevant citations were retrieved from PubMed and Embase, and 178 of them were excluded during the review of titles and abstracts. Four articles remained to be fully evaluated. Nevertheless, following careful analysis of their full texts, one article was excluded because it did not have a control group. Therefore, three articles (Lindholm *et al.*, 2004; Tiwari *et al.*, 2009; de Souza *et al.*, 2015) plus the present case-control study were included in our meta-analysis, totalizing four articles (five studies). In total, 717 controls without DKD and 648 cases with this complication were analyzed for the -866G/A polymorphism, and 937 controls and 857 cases for the Ins/Del polymorphism. The article by Tiwari *et al.* (2009) analyzed the two *UCP2* polymorphisms in two different populations from South India and North India, and, because of that, their results are shown separately.

With exception of the present case-control study, the other three articles included only T2DM patients. Two studies comprised Caucasian populations (Lindholm *et al.*, 2004; de Souza *et al.*, 2015), the present study investigated a mixed population, while Tiwari *et al.* (2009) analyzed two Asian populations. All studies investigated the Ins/Del polymorphism, while the study by Lindholm *et al.* (2004) was the only one that did not investigate the -866G/A polymorphism. Two studies (Lindholm *et al.*, 2004; de Souza *et al.*, 2015) plus the present case-control classified DKD using the UAE, while one study (Tiwari *et al.*, 2009) classified DKD using serum creatinine levels. Genotype and allele distributions of the *UCP2* polymorphisms in case and control samples from the different studies, as well as their respective ORs (95% CI) for association with DKD, are shown in Table S3. Quality assessment using the NOS scale showed that most studies were considered as having good quality since 8 stars were given for the studies by Lindholm *et al.* (2004) and Souza *et al.* (2015), and 7 stars for the study by Tiwari *et al.* (2009).

Table 4 summarizes the results of quantitative pooled analyses for associations between -866G/A and Ins/Del polymorphisms and susceptibility to DKD. Our results showed no significant associations between these polymorphisms and DKD under allele contrast, additive, recessive, or dominant inheritance models. A significant heterogeneity was observed among studies of the -866G/A polymorphism considering the dominant model of inheritance (Table 4). Thus, sensitivity analyses were performed to evaluate the effect of each individual study on the meta-analysis performed for this model. This was carried out by repeating the meta-analysis excluding a different study at a time. These analyses showed that the study by Tiwari *et al.* (2009) explained the observed heterogeneity in the meta-analysis of the -866G/A polymorphism under a dominant model. However, after exclusion of this study from the respective meta-analysis, the pooled OR did not remain significant (OR= 0.91, 95% CI 0.71 – 1.16).

**Table 4:** Pooled measures for associations between the *UCP2* -866G/A and Ins/Del polymorphisms and susceptibility to DKD.

Inheritance model	*n* studies	*n* controls	*n* cases	I^2^ (%)	Pooled OR (95% CI)
*UCP2 -866 G/A*					
Allele contrast^b^	4	717	648	46.0	1.03 (0.88-1.21)
Additive^b^	4	390	351	0.0	1.04 (0.75-1.45)
Recessive^b^	4	717	648	0.0	1.05 (0.78-1.42)
Dominant^a^	4	717	648	53.2	1.04 (0.74-1.45)
*UCP2 Ins/Del*					
Allele contrast^b^	4	719	641	0.0	0.96 (0.81-1.14)
Additive^b^	4	444	413	0.0	1.08 (0.71-1.63)
Recessive^b^	4	719	641	13.3	1.11 (0.74-1.65)
Dominant^b^	5	937	857	0.0	0.89 (0.74-1.08)

^a^ If significant heterogeneity was detected (I^2^ > 50%), the DerSimonian and Laird random effect model (REM) was used to calculated OR (95% CI); ^b^ if heterogeneity was not significant, the fixed effect model (FEM) was used for this calculation. DKD: diabetic kidney disease.

## Discussion

ROS overproduction is one of the main mechanisms by which hyperglycemia leads to chronic diabetic complications, including DKD (Brownlee, 2005; Rich, 2006; Giacco and Brownlee, 2010). Although UCP2 has a recognized role in reducing oxidative stress, to date, only few studies have evaluated the association between polymorphisms in the *UCP2* gene and DKD. Therefore, aiming to better understand the relationship between the *UCP2* -866G/A and Ins/Del polymorphisms and the development of this chronic diabetic complication, we performed a case-control study and a meta-analysis of genetic association studies on this subject.

It is well known that functional polymorphisms might influence gene expression and regulate the final quantity of the corresponding protein in a given tissue. Among the three common polymorphisms more studied in the *UCP2* gene, only the -866G/A polymorphism is clearly functional. This polymorphism is located in the *UCP2* promoter region and alters an important binding site of transcription factors; therefore, increasing or decreasing *UCP2* expression according to the binding of tissue-specific transcription factors (Oberkofler *et al.*, 1997; Cassell *et al.*, 1999; Esterbauer *et al.*, 2001; Wang *et al.*, 2004; de Souza *et al.*, 2012). Although there is no evidence that the Ins/Del polymorphism has a functional impact on *UCP2* expression, it is located in the 3’ untranslated region of the gene. This region is the main site for ligation of microRNAs, which are major regulators of gene expression (Assmann *et al.*, 2018). Thus, the Ins/Del polymorphism might change a ligation site for some microRNAs. Indeed, in a previous study, we used a bioinformatics analysis to show that several predicted interaction regions with microRNAs were found in the *UCP2* 3’ untranslated region. However, only one microRNA (hsa-miR-3668) strongly targeted the sequence where the Ins/Del polymorphism is located. The 45 bp Ins allele disrupts the ligation site for this miRNA; thus, probably changing *UCP2* expression (de Souza *et al.*, 2015). The Ala55Val polymorphism causes a conservative amino acid change and there is no indication that it causes a functional change in the protein.

Our case-control study suggested that both analyzed polymorphisms and the haplotypes constituted by them are not associated with DKD in T1DM patients. In contrast, our previous study showed that the polymorphic -866A/55Val/Ins haplotype was associated with DKD in Brazilian T2DM patients after adjustment for age, gender, treatment with ACE-inhibitors, triglycerides, and estimated GFR levels (de Souza *et al.*, 2015). In both studies, DKD was classified using UAE levels. Souza *et al.* (2015) also reported that T2DM patients carrying the -866A/55Val/Ins haplotype (dominant model) showed lower estimated GFR compared to patients with the reference haplotype, which was not observed in the present study. These discrepancies may be explained by differences in DKD pathophysiology between T1DM and T2DM (Ruggenenti and Remuzzi, 2000). T1DM is caused by autoimmune destruction of pancreatic beta-cells, leading to the total absence of insulin secretion and, consequently, hyperglycemia. As already mentioned, hyperglycemia leads to the activation of glucose-dependent pathways, such as advanced glycation end-products, protein kinase C, polyol and hexosamine. Excessive activation of these pathways causes the accumulation of their substrates, cellular dysfunction, inflammation, apoptosis, and fibrosis in renal cells exposed to excessive glucose flux (Thomas *et al.*, 2015; Katsarou *et al.*, 2017). In contrast, T2DM is caused by obesity-induced insulin resistance associated with a relative decrease in insulin secretion. Therefore, besides the activation of glucose-dependent pathways, as occurs in T1DM, DKD in T2DM patients is also influenced by obesity, hypertension and dyslipidemia (Thomas *et al.*, 2015) (Figure 1).

Also, we cannot fully exclude the possibility of false-negative results when analyzing associations between the *UCP2* polymorphisms and DKD. Although we had more than an 80% power (α = 0.05) to detect an OR = 2.0 for the association with the -866G/A and Ins/Del polymorphisms, we cannot rule out the possibility that these polymorphisms would be individually associated with DKD with lower ORs. There is also a possibility that these two polymorphisms are only associated with DR, an association observed in both T1DM and T2DM patients (Crispim *et al.*, 2010). Considering that the majority of DKD patients have some degree of DR (Scheffel *et al.*, 2004), it is plausible that the association with DKD in T2DM patients (de Souza *et al.*, 2015) was not independent of DR.

Meta-analysis has been regarded as a powerful method for pooling data from different studies because it could overcome the problem of small sample sizes, as well as insufficient statistical power of genetic association studies for common diseases (Stroup *et al.*, 2000). Therefore, trying to overcome the problem of small sample size, we also performed a meta-analysis that included three published studies from different populations plus the results from the present case-control study. Meta-analysis results indicated that the -866G/A and Ins/Del polymorphisms are not associated with DKD. Among the studies included in our meta-analysis, only the study by Tiwari *et al.* (2009) showed an association between the -866G/A polymorphism and DKD in a population from southern India. These authors did not observe an association between this polymorphism and DKD in a population from northern India. The other studies were not able to show an association of the -866G/A or Ins/Del polymorphisms with DKD, including the study by de Souza *et al.* (2015) that, as already mentioned, only observed an association with the disease when analyzing the haplotypes constituted by the two polymorphisms.

Rudofsky *et al.* (2006, 2007) also observed that the frequency of DKD was similar among German T1DM (Rudofsky *et al.*, 2006) and T2DM (Rudofsky *et al.*, 2006, 2007) patients carrying the different genotypes of the -866G/A polymorphism. These two studies were not included in our meta-analysis because they did not include an appropriate control group. In addition, Tripathi *et al.* (2008) reported an association between the Ins/Del polymorphism and risk for end-stage renal disease in non-diabetic subjects from northern India; however, genotype distributions of this polymorphism were not in Hardy-Weinberg Equilibrium in the control group. Thus, this study could not be included in our meta-analysis and should be interpreted with caution.

Therefore, to date, most studies indicated that the -866G/A and Ins/Del polymorphisms are not risk factors for DKD. We acknowledge that certain factors unrelated to the *UCP2* polymorphisms could have interfered with the present findings. First, meta-analysis is prone to publication bias, and although we have attempted to trace unpublished observations, we cannot be sure that smaller negative studies were overlooked. Second, although the meta-analysis increased the statistical power, the total sample power might still not be sufficient to show associations with lower ORs. Third, heterogeneity is potentially a significant problem when interpreting the results of any meta-analysis, and our meta-analysis showed significant inter-study heterogeneity when analyzing the -866G/A polymorphism in the dominant model of inheritance. The exclusion of the study by Tiwari *et al.* (2009) was able to reduce heterogeneity; however, this exclusion did not change the association with DKD. Therefore, we could not fully exclude the possibility that the heterogeneity observed might reduce our power to detect true associations.

Despite these negative results regarding associations between *UCP2* polymorphisms and DKD, functional studies have suggested that changes in *UCP2* expression play an important role in the development of renal damage. Qiu *et al.* (2012) reported that oral administration of genipin, a UCP2 inhibitor, partially prevented the progression of DKD in C57BL/6J mice by reducing glucose-induced albumin leakage through podocyte monolayers, consequently improving podocyte function. Accordingly, Jiang *et al.* (2013) showed that UCP2 was induced in kidney tubular epithelial cells after unilateral ureteral obstruction in mice, while those mice with ablated *UCP2* resisted obstruction-induced kidney fibrosis. Moreover, *UCP2* knockdown in NRK-52E tubular cells abolished the effect of TGF-β1 treatment, decreasing extracellular matrix production (Jiang *et al.*, 2013). In contrast, Chen *et al.* (2014) demonstrated that inhibition of UCP2 by genipin increased oxidative stress in rat proximal tubular cells treated with high glucose medium, and this led to increased cell apoptosis. *UCP2* knockdown in renal mesangial cells of rats also increased oxidative stress, inflammation, and apoptosis *in vitro* (Di Castro *et al.*, 2013). Therefore, whether UCP2 has a protective or deleterious effect in renal function remains to be clarified.

In conclusion, data reported here suggest that the *UCP2* -866G/A and Ins/Del polymorphisms are not important risk factors for DKD, classified according to UAE values. Further additional studies with large sample sizes are necessary to elucidate the effects possibly played by *UCP2* polymorphisms in the pathogenesis of DKD.

## Supplementary material

The following online material is available for this article:

MeSH terms used for searching articles to be included in meta-analysis.

Table S1 Genotype and allele frequencies of *UCP2* -866G/A and Ins/Del polymorphisms. in T1DM patients.

Table S2 Genotype and allele frequencies of *UCP2* -866G/A and Ins/Del polymorphisms in T1DM patients and in nondiabetic subjects.

Table S3 Genotype and allele distributions of the *UCP2* -866G/A and Ins/Del polymorphisms in T1DM patients with (cases) and without (controls) DKD.
