# Supplementary material for: -866G/A and Ins/Del polymorphisms in the UCP2 gene and diabetic kidney disease: case-control study and meta-analysis
Source: Genet Mol Biol. 2020 Mar 27;43(2):e20180374. doi: 10.1590/1678-4685-GMB-2018-0374 (PMC7198021; doi:10.1590/1678-4685-GMB-2018-0374)
Supplement: Supplementary file 1 [file 1415-4757-GMB-43-2-e20180374-suppl1.pdf]

Supplementary Material to "866G/A and Ins/Del polymorphisms in the UCP2 gene and diabetic kidney disease: case-control study and meta-analysis"

Medical Subject Heading (MeSH) terms used for searching articles to be included in meta-analysis: ("diabetes mellitus" OR "diabetes mellitus, type 1" OR "diabetes mellitus, type 2" OR "diabetic nephropathy" OR "diabetes complications") AND ("polymorphism, genetic" OR "polymorphism, single-stranded conformational" OR "polymorphism, single nucleotide" OR "polymorphism, restriction fragment length" OR "amplified fragment length polymorphism analysis" OR "DNA copy number variations" OR "mutation" OR "frameshift mutation" OR "mutation rate" OR "INDEL mutation" OR "mutation, missense" OR "point mutation" OR "codon, nonsense") AND ("uncoupling protein 2").
