# Supplementary material for: -866G/A and Ins/Del polymorphisms in the UCP2 gene and diabetic kidney disease: case-control study and meta-analysis
Source: Genet Mol Biol. 2020 Mar 27;43(2):e20180374. doi: 10.1590/1678-4685-GMB-2018-0374 (PMC7198021; doi:10.1590/1678-4685-GMB-2018-0374)
Supplement: Supplementary file 2 [file 1415-4757-GMB-43-2-e20180374-suppl2.pdf]

Supplementary Material to "866G/A and Ins/Del polymorphisms in the UCP2 gene and diabetic kidney disease: case-control study and meta-analysis"

Table S1 - Genotype and allele frequencies of *UCP2* -866G/A and Ins/Del polymorphisms in T1DM patients with UAE <30 mg/24h (T1DM control), T1DM patients with UAE 30-300 mg/24h (moderate DKD), and T1DM with UAE >300 mg/24h (severe DKD).

**Polymorphisms**	**T1DM control group**	**Moderate DKD**	**Severe DKD**	***P****
**- 866G/A (rs659366)**	n = 223	n = 110	n = 52	
Genotype				
G/G	77 (34.5)	42 (38.2)	19 (36.5)	0.962
G/A	107 (48.0)	48 (43.6)	24 (46.2)	
A/A	39 (17.5)	20 (18.2)	9 (17.3)	
Allele				
G	0.59	0.60	0.60	0.929
A	0.41	0.40	0.40	
Recessive model				
G/G + G/A	184 (82.5)	90 (81.8)	43 (82.7)	0.985
A/A	39 (17.5)	20 (18.2)	9 (17.3)	
Additive model				
G/G	77 (66.4)	42 (67.7)	19 (67.9)	0.978
A/A	39 (33.6)	20 (32.3)	9 (32.1)	
Dominant model				
G/G	77 (34.5)	42 (38.2)	19 (36.5)	0.803
G/A + A/A	146 (65.5)	68 (61.8)	33 (63.5)	
**Ins/Del**	n = 222	n = 103	n = 53	
Genotype				
Del/Del	107 (48.2)	57 (55.3)	25 (47.2)	0.768
Ins/Del	93 (41.9)	36 (35.0)	23 (43.4)	
Ins/Ins	22 (9.9)	10 (9.7)	5 (9.4)	

**Polymorphisms**	**T1DM control group**	**Moderate DKD**	**Severe DKD**	***P****
Allele				
Del	0.69	0.73	0.69	0.608
Ins	0.31	0.27	0.31	
Recessive model				
Ins/Del + Del/Del	200 (90.1)	93 (90.3)	48 (90.6)	0.994
Ins/Ins	22 (9.9)	10 (9.7)	5 (9.4)	
Additive model				
Del/Del	107 (82.9)	57 (85.1)	25 (83.3)	0.929
Ins/Ins	22 (17.1)	10 (14.9)	5 (16.7)	
Dominant model				
Del/Del	107 (48.2)	57 (55.3)	25 (47.2)	0.442
Ins/Del + Ins/Ins	115 (51.8)	46 (44.7)	28 (52.8)	
**Presence of the *UCP2* mutated haplotype**	**n = 209**	**n = 101**	**n = 49**	
0 or 1 mutated allele	110 (52.6)	59 (58.4)	24 (49.0)	0.805
2 mutated alleles	63 (30.1)	27 (26.7)	17 (34.7)	
3 or 4 mutated alleles	36 (17.2)	15 (14.9)	8 (16.3)	

Data are shown as number (%) or proportion. DKD: diabetic kidney disease; T1DM: type 1 diabetes mellitus; UAE: urinary albumin excretion. **P-values* were calculated using Chi-square tests.
