# Supplementary material for: -866G/A and Ins/Del polymorphisms in the UCP2 gene and diabetic kidney disease: case-control study and meta-analysis
Source: Genet Mol Biol. 2020 Mar 27;43(2):e20180374. doi: 10.1590/1678-4685-GMB-2018-0374 (PMC7198021; doi:10.1590/1678-4685-GMB-2018-0374)
Supplement: Supplementary file 3 [file 1415-4757-GMB-43-2-e20180374-suppl3.pdf]

Supplementary Material to "866G/A and Ins/Del polymorphisms in the UCP2 gene and diabetic kidney disease: case-control study and meta-analysis"

Table S2 - Genotype and allele frequencies of *UCP2* -866G/A and Ins/Del polymorphisms in T1DM patients and nondiabetic subjects.

Polymorphisms	Nondiabetic subjects	T1DM patients	*P*-value*	Adjusted OR (95% CI) /† *P*-value
**-866G/A**	n = 489	n = 385		
Genotype				
G/G	187 (38.2)	138 (35.8)	0.754	1
G/A	221 (45.2)	179 (46.5)		1.050 (0.748 – 1.476)/ 0.777
A/A	81 (16.6)	68 (17.7)		1.123 (0.713 – 1.770)/ 0.617
Allele				
G	0.61	0.59	0.459	-
A	0.39	0.41		
Recessive model				
G/G + G/A	408 (83.4)	317 (82.3)	0.735	1
A/A	81 (16.6)	68 (17.7)		1.094 (0.722 – 1.657)/ 0.673
Additive model				
G/G	187 (69.8)	138 (67.0)	0.584	1
A/A	81 (30.2)	68 (33.0)		1.117 (0.708 – 1.762)/ 0.634
Dominant model				
G/G	187 (38.2)	138 (35.8)	0.511	1
G/A + A/A	302 (61.8)	247 (64.2)		1.069 (0.777 – 1.471)/ 0.681

**Ins/Del**	n = 374	n = 378		
Genotype				
Del/Del	194 (51.9)	189 (50.0)	0.837	1
Ins/Del	147 (39.3)	152 (40.2)		1.111 (0.787 – 1.568)/ 0.550
Ins/Ins	33 (8.8)	37 (9.8)		1.173 (0.660 – 2.087)/ 0.586
Allele				
Del	0.72	0.70	0.545	-
Ins	0.28	0.30		
Recessive model				
Ins/Del + Del/Del	341 (91.2)	341 (90.2)	0.742	1
Ins/Ins	33 (8.8)	37 (9.8)		0.121 (0.643 – 1.954)/ 0.687
Additive model				
Del/Del	194 (85.5)	189 (83.6)	0.682	1
Ins/Ins	33 (14.5)	37 (16.4)		1.169 (0.660 – 2.071)/ 0.593
Dominant model				
Del/Del	194 (51.9)	189 (50.0)	0.660	1
Ins/Del + Ins/Ins	180 (48.1)	189 (50.0)		1.123 (0.811 – 1.555)/ 0.486

Data are shown as number (%) or proportion. T1DM: type 1 diabetes mellitus. **P*-values were calculated using Chi-square tests. † *P*-values and OR (95% CI) obtained using logistic regression analyses adjusting for hypertension and ethnicity.
