# Supplementary material for: -866G/A and Ins/Del polymorphisms in the UCP2 gene and diabetic kidney disease: case-control study and meta-analysis
Source: Genet Mol Biol. 2020 Mar 27;43(2):e20180374. doi: 10.1590/1678-4685-GMB-2018-0374 (PMC7198021; doi:10.1590/1678-4685-GMB-2018-0374)
Supplement: Supplementary file 4 [file 1415-4757-GMB-43-2-e20180374-suppl4.pdf]

Supplementary Material to “866G/A and Ins/Del polymorphisms in the UCP2 gene and diabetic kidney disease: case-control study and meta-analysis”

Table S3 - Genotype and allele distributions of the *UCP2* -866G/A and *UCP2* Ins/Del polymorphisms in DM patients with (cases) and without (controls) DKD.

-866G/A polymorphism		Cases (n) by total and genotype				Controls (n) by total and genotype				G allele frequency (%)		
Reference, year	Ethnicity	Total	G/G	G/A	A/A	Total	G/G	G/A	A/A	Cases	Controls	OR (95% CI) [a]
Tiwari, *et al*. 2009[a]	Asian	106	43	52	11	146	80	56	10	65.1	74.0	1.524 (1.037 – 2.239)
Tiwari, *et al.* 2009[b]	Asian	90	43	37	10	75	30	36	9	68.3	64.0	0.824 (0.521 – 1.302)
Souza, *et al*., 2015	Caucasian	287	101	134	52	278	99	131	48	58.5	59.2	1.027 (0.810 – 1.301)
The present case-control study	Caucasian	165	62	74	29	218	74	104	40	60.0	59.0	0.913 (0682 – 1.221)
Ins/Del polymorphism		Cases (n) by total and genotype				Controls (n) by total and genotype				Del allele frequency (%)		

Reference	Ethnicity	Total	Del/Del	Ins/Del	Ins/Ins	Total	Del/Del	Ins/Del	Ins/Ins	Cases	Controls	OR (95% CI)*
Lindholm, *et al.*, 2004	European	216	NS	NS	NS	218	NS	NS	NS	NS	NS	NA
Tiwari, *et al.*, 2009[a]	Asian	105	73	30	2	149	108	38	3	83.8	85.2	1.115 (0.685 – 1.815)
Tiwari, *et al.*, 2009[b]	Asian	90	61	26	3	75	45	24	6	82.0	76.0	0.685 (0.400 – 1.169)
Souza, *et al.*, 2015	Caucasian	287	144	110	33	278	132	124	22	70.0	69.8	1.021 (0.792 – 1.316)
The present case-control study	Caucasian	159	82	62	15	217	106	89	22	71.0	69.0	0.921 (0.671 – 1.265)

* Calculated from the available genotypes. DM: diabetes mellitus; DKD: diabetic kidney disease; NS, not shown; NA, not available.
